# Participant and researcher understandings of research responsibilities in malawi: a comparative analysis

**DOI:** 10.1186/s12910-025-01306-1

**Published:** 2025-10-24

**Authors:** Gertrude Mwase Banda, Blessings M. Kapumba, Wezzie Nyapigoti, Deborah Nyirenda, Nicola Desmond, Lucinda Manda-Taylor

**Affiliations:** 1 Department of Health Systems and Policy, Kamuzu University of Health Sciences, Blantyre, Malawi; 2 Malawi Liverpool Wellcome Programme, Blantyre, Malawi; 3 Liverpool School of Tropical Medicine, Liverpool, UK

**Keywords:** Responsibilities, Roles, Clinical research, Medical research, Research participation, Ethics, Research engagement

## Abstract

**Purpose:**

Current literature seldom discusses the functions and duties of individuals participating in medical research and the importance they attribute to their research involvement. To fill this void, our study investigated participants’ perceptions of their roles and responsibilities in the research context and compared these perceptions with those of research staff.

**Methods:**

A cross-section qualitative study with 21 semi-structured interviews with participants purposefully selected from clinical and non-clinical trials, and research staff. We also conducted two focus group discussions: one with participants from clinical trials and one from non-clinical trials. We analysed data using thematic analysis methods.

**Results:**

The responsibilities of research participants are understood differently between research participants and research staff. Two broad themes emerged from the findings: relational responsibilities of research participants and functional responsibilities of participants.

**Conclusion:**

There are differences in perceived responsibilities between researchers and participants, and this disconnect needs to be recognised. Researchers should focus on how best to communicate responsibilities to enhance awareness and achieve mutual understanding.

**Supplementary Information:**

The online version contains supplementary material available at 10.1186/s12910-025-01306-1.

## Introduction

Clinical research plays a crucial role in advancing medical knowledge and improving healthcare outcomes [1]. The backbone of conducting high-quality clinical research is the enrollment and retention of participants who understand the clinical research processes [2]. Within the ever-evolving realm of clinical research, the point at which participants’ expectations intersect with the pragmatic aspects of their roles and responsibilities emerges as a pivotal focal point that merits investigation.

Ethical considerations in research encompass both researchers and participants, with a focus on the responsibilities and rights of participants. The Council for International Organizations of Medical Sciences (CIOMS) states that research participants can withdraw at any time without giving any reasons [3]. However, discussions surrounding the ethical duties of research participants have been limited [4]. Physicians involved in medical research must prioritize protecting participants’ well-being, emphasising that the responsibility for safeguarding research participants always lies with healthcare professionals, not the participants themselves [5] Researchers play a pivotal role in identifying both the ethical issues and their associated risks and benefits, and in formulating procedures to address the problems identified [4].

While informed consent is a cornerstone of ethical biomedical research, it is acknowledged that consent forms may have limitations regarding how participants interpret and understand the content [4]. The researcher must inform the participant about what is being studied, the risks and benefits associated with participation, their right to withdraw at any time, compensation, and where to lodge concerns, if any [6]. Interestingly, the responsibilities assumed by participants in research are often absent from consent forms, and the guidelines regarding the content of consent forms do not typically include participants’ responsibilities as a core component. Thus, the participant’s perspective on their responsibilities in research is an aspect that warrants further exploration [7]. Through summarizing a paper on participants’ responsibilities in research, the responsibilities of research participants can be defined as the obligations they undertake when they decide to enrol in research, which are based on duties related to promise-keeping, avoiding harm to oneself or others, beneficence, and reciprocity [8].

However, the ethical dimension of research primarily focuses on the researcher’s responsibilities, with limited attention given to the responsibilities and understanding of participants [4]. Research should be viewed as a partnership between participants and investigators, where both parties know their responsibilities. Participants who understand their responsibilities may be more assertive [8], committed, adhere to research protocols, and ultimately promote study retention. Despite the importance of this perspective, literature on participants’ responsibilities in research still needs to be explored [8]. This study aimed to answer the following questions: What do research participants perceive as their responsibility in research and how does that compare with research staff perceptions? What do they know as their responsibility in research? What are the enablers to their understanding? What perceptions do they have towards their responsibilities? This paper presents results on one question: How do participants and research staff differ in their perceptions of research responsibilities?

## Methodology

### Study design

This was a cross-sectional research study using qualitative approaches to explore the views of research participants and research staff on the responsibilities of research participants.

### Study setting

The study was conducted at Queen Elizabeth Central Hospital (QECH) in Blantyre District and Chikwawa District Hospital (CK DHO) in Chikwawa, both located in the southern region of Malawi. Public hospitals account for most of the care provided through the healthcare system in Malawi [9]. Hospitals are arranged into three levels of health care: tertiary, which consists of central hospitals; secondary, which accounts for district hospitals; and primary level, which consists of health centres and provides basic care. QECH, a tertiary hospital and major referral hospital, receives patients from 9 districts in Southern Malawi and occasional referrals from across the country. QECH hosts substantial clinical studies and is located near two research institutions and a university. CK DHO operates as a secondary-level healthcare facility with a 230-bed capacity, catering to a population of 628,282 people. The hospital also supports a substantial number of research studies. We included QECH and CK DHO sites to reach a broader population. Non-CTs in this research context referred to studies that did not involve any interventions or control groups.

### Sample size and data collection

Thirty-five potential research participants and eight research staff were screened for participation in in-depth interviews (IDIs) or focus group discussions (FGDs). Twenty-one individuals were purposefully recruited for IDIs, while six were not recruited due to time constraints or ineligibility. The final sample consisted of fifteen research participants and six research staff members, providing diverse perspectives for rich data collection. The sample size for research participants aligns with the literature on data saturation (10,11). The sample size for the research staff was determined to be adequate for data saturation, in line with [10], which states that six interviews are sufficient to allow for the development of themes and useful interpretations. Although the sample was relatively homogeneous, researchers sought to explore the range of perceptions regarding roles and responsibilities in research. We also recruited 16 participants for Focus group discussions (FGDs) divided into two groups of 8 in each group (eight participating in clinical trials and eight in non-clinical trials studies). Eligibility criteria for participation in the research were participants over 18 years of age, willing to participate, able to provide informed consent, and attending the hospital where the research was being conducted. Participants who attended IDIs did not participate in FGDs. Participants were drawn from clinical trials and non-clinical trials that were actively recruiting after obtaining permission to include them in our study population. We asked research staff working in the studies to inform potential participants of the possibility of being recruited into our study at or soon after their exit visit. During their exit visit, interested participants were screened for eligibility, briefed about the study, and given a study information leaflet to review at home and consult with significant others regarding their participation. They were then invited to participate in a consent process and interviews seven days after the screening. Fifteen individual interviews were conducted with research participants and six research staff to collect rich data. We also conducted two focus group discussions with participants in both clinical trials and non-clinical trials. We used both individual interviews and focus group discussions to achieve a more nuanced understanding of the topic [11]. Data collection ended when saturation was achieved. Saturation was reached after the interviews, and no new information was produced. The study was conducted over 12 months, from December 2021 to November 2022, with data collection concluding in March 2022. Data was collected using semi-structured interview topic guides with open-ended questions (S1 appendices). The individual interview guide was piloted with two participants, and a few changes were made. The pilot data was excluded from the final analysis. The research staff guide was not piloted because the authors believed that all issues had been resolved with the guide based on the research participants’ pilot.

The focus group discussion guide was not piloted due to challenges in gathering everyone together, which were exacerbated by complications with COVID, thereby reducing the pool of potential participants. All interviews were conducted by the principal investigator (PI) and an experienced Research Assistant in qualitative data collection. Topics included what participants understand as their role in research. Interviews and FGDs were conducted in a private room within each of the hospitals. Interviews were conducted in the language of the participant’s choice, either Chichewa or English. Participants were encouraged to provide comprehensive information using open-ended questions and probes, and to establish rapport during the initial contact. All interviews were audio recorded and later translated and transcribed verbatim. Each interview lasted approximately an hour, with the focus group session lasting 90 min.

### Data analysis

Data was analysed thematically. Transcripts were read and reread to allow familiarization with the dataset. Transcripts were continually compared to identify common views. Research staff accounts were compared with those of research participants to observe differences and similarities in their perceptions. Coding was done both manually and in NVivo 12 software. Codes were then generated inductively and were data-driven, allowing the emergence of new themes. The generated codes were later categorized, so similar codes were combined and then grouped to identify common patterns. The classified and grouped codes were then arranged into initial themes. Common patterns and concepts were identified within each theme. Relationships and connections were examined with an open mind, allowing for the possibility of changing patterns. A review of the themes was then conducted, resulting in the definition and naming of the themes. We employed an iterative process. As researchers analysed the data, they continued to identify emerging themes and patterns, using these to refine the data collection and focus on specific areas that required further exploration. This process continued until no significant themes or patterns emerged, and data saturation was reached.

### Ethics consideration

The College of Medicine approved the study Research Ethics Committee (COMREC) protocol number P.04.21/3311 and the Liverpool School of Tropical Medicine Research Ethics (LSTM) Committee protocol number 21–038. Additional approval was obtained from the QECH research ethics committee, the Blantyre District Health Office (DHO) and the Chikwawa DHO. Informed consent was obtained from all participants. The researchers informed all participants that they had the right to withdraw without giving reasons and that refusing to participate would not affect the care they received at the hospital.

## Results

The tables below present the sociodemographic characteristics of the study participants. In total, 37 participants were recruited, with a mean age of 41 years for research staff and 39 years for research participants. Tables 1 and 2 provide detailed demographic characteristics for each group.

**Table 1:** Demographic characteristics of research participants

Characteristic	Total number
Gender Male Female	22 15
Educational level Primary Secondary Tertiary None	12 9 14 2
Employment status Employed Unemployed Self-employed Student Retire	17 5 10 3 2

**Table 2:** Demographic characteristics of research staff

Characteristic	Total number
Gender Male Female	3 3
Educational level Tertiary	6
Employment status Employed	6
Designation Research nurses Field research assistants Principal Investigators	2 2 2

### Overview of results

Two broad themes emerged after the analysis of results: perceptions of responsibilities as relational and perceptions of responsibilities as functional. The duties of the participants mentioned by participants were more relational, acknowledging the complementary roles between research staff and study participants. At the same time, those reported by research staff were more functional, study- or protocol-related. Table 3 is a summary of themes that emerged from this study.

**Table 3:** A summary of themes and subthemes

Themes	Subthemes
Perceptions of responsibilities as relational	• Help researchers achieve research objectives • Advocating for researchers • Motivating others
Perceptions of responsibilities as functional	• Responsibilities are dependent on study design and requirements. • Responsibilities are dependent on research participant.

### Perceptions of responsibilities

#### Knowledge and Understanding of participants’ responsibilities

Issues surrounding knowledge and understanding of research participant responsibility brought diverse views. This study found differences in perceptions amongst groups of interviewees. For study participants, their knowledge and sense of responsibility were shaped by their experience participating in investigations, rather than by the information provided by research staff. Most of them acknowledged the need for more details about their duties from researchers.

*‘They did not tell me*,* they didn’t say. I did ask*,* but they said that since we will continue to engage with you*,* we will let you know. And that was last year.’ (Study participant*,* 010 NCT)*.

*‘I have never been told anything about that*,* saying your roles are this and that*,* no. The roles I have explained to you are the ones I know in my heart. But to say I was once called over to be told my roles…no.’ (Study participant*,* 005 CT)*.

Despite the unavailability of information on responsibilities from research staff, study participants assumed roles and responsibilities based on their understanding of their contribution to the research. Their perception of duties was based on the knowledge that they have a part to play in making research work, emphasising relational responsibilities.

A Perceived relational responsibilities
  Participants emphasised relational responsibilities, which are self-assigned or self-given responsibilities aimed at building relationships with research staff, maintaining participation, and avoiding disappointment to research staff. They emphasised their role in encouraging others to volunteer, dispelling myths surrounding research, and helping researchers achieve objectives.
  i. Help achieve research objectives.
    Participants felt they were helping researchers fulfil the study’s aims by participating in research. They believe that researchers’ work would be incomplete without participants. Research participants believe they play a crucial role by providing researchers with data and a platform to accomplish their planned work, thereby helping to achieve research objectives.
    *‘To help researchers understand what they are looking at*,* whether medicine or disease*,* the efficacy of a drug in treating a certain disease*,* or anything about a disease. So*,* that is how we play our role. So*,* I feel we have a role to help researchers accomplish their work.’ (Study participant*,* CT FGD)*.
    Research participants felt indebted to the research staff due to the perceived benefits they received during their participation in the study. They took on the role of helping as advocates for the research staff.
  ii. Advocating for researchers
    Clinical research requires a trusting relationship between research staff and the communities in which it occurs or where research participants are recruited. Research staff are responsible for gaining the trust of the public and the communities in which they conduct research. However, participants felt that one of their roles in research was to dispel rumours surrounding research prevalent in their communities, thereby helping establish research trustworthiness before a trusting relationship with research staff was built.
    *‘We wanted to go and see where they dispose of the blood because the people were having difficulty with blood samples. When we told the researchers*,* they agreed*,* and we went to their office. We had a meeting down here*,* and after the meeting*,* we visited the lab. When we returned*,* we assured the community that we handle blood properly and that we have seen where it is disposed of*,* ensuring it is properly disposed of and not reused. There is no problem; they use blood as a sample to conduct tests*,* and after that*,* they dispose of it. They do not use it in any other way. In their minds*,* they thought that maybe researchers sell the blood*,* but after our explanation and assurance*,* they took the courage to participate*,* and we were together for the five years that the study was running.’ (Study participant 011*,* NCT)*.
    Research participants believed myths surrounding research discourage many people in the community from participating in research. Research participants felt a duty to inform others that there is nothing harmful about research, having gone through it themselves. This is a self-assigned responsibility, and participants never felt like they were doing this role on behalf of researchers. They do this to pay back and not disappoint research staff, thereby maintaining the researcher-participant relationship.
  iii.  Motivating others
    As stated above, the motivation to participate in research stemmed from several drivers: seeking help, helping researchers, and gaining insight into medical research processes were true for some research participants. Some said that by explaining the benefits and positive research experience to people in their circles, they encourage other community members who need clarification about participating in a study to volunteer in research. Participants indicated that motivating others to volunteer in research was one of their roles.
    ‘*…Some people approach me and say they have been invited to participate in a research study. Therefore*,* my role is to encourage such individuals by advising them not to refuse; they will likely find two or three things that will benefit their health. So*,* I am there to encourage them.*’*(Study Participant*,* 014 NCT)*.
    This was largely dependent on what they considered their motivation for participating in research. It was mainly dependent on the benefits they accessed while participating or because they experienced it..
B Perceived functional responsibilities of participants
  While study participants emphasised relational responsibilities, research staff participating in the study highlighted the functional responsibility of participants. They mentioned, for example, understanding the study, fulfilling study visits, providing biological samples, adhering to medication and other instructions given by study staff, and being committed to the study requirements to meet their end of the bargain once they agree to participate. The study design and protocol drove the responsibilities mentioned by the researchers. In essence, researchers believe that responsibilities are not standardized; they vary based on the study design and protocol. They said that the duties of participants depend on what is stipulated in the study protocol. In addition, researchers also believed that the study participants’ responsibilities depended on how they perceived the trial risks and how they could protect themselves and their families.
  i. Responsibilities are dependent on the study design and requirements
    Research staff indicated that the responsibilities assumed by research participants when they enrol in research depend on the information provided by research staff at the point of recruitment, specifically during the informed consent process. Information is based on the participant information leaflet and the informed consent form. The responsibilities of research participants vary over time and primarily depend on the study’s requirements.
    *The role of a person participating in research depends on the information you have given them about the study. Let’s say*,* for example*,* that research requires two to three visits*,* including adherence to visits and medicines. Then*,* you must explain to the person the roles they need to perform. If there is a need for follow-up*,* then you must explain to the person so that when he is aware of the dates*,* samples to be collected…’ (Research staff*,* 04)*.
    In addition, research staff felt that nothing surpasses a conversation with a potential research participant to help them understand their role in research. The interface between the study participant and researcher during the informed consent process provides the foundation for informing study participants of their responsibilities by discussing the participant information leaflet, which contains all the information regarding the participant’s responsibilities.
    *‘And for me, the time that you talk to a potential participant is the most rewarding in the research process in getting a participant to fully understand what their roles would be in the study or the research and, therefore, we spend much time explaining what we plan to do, or we are doing in the research, and we hope and expect that the participant understands that before they commit to taking part in the research. Therefore, their role is really to ensure that they understand what the research is about and, therefore, behave in a way that will contribute to achieving the intended outcomes of that research because if participants don’t understand and if you have a study where you have a follow-up, it’s when you get participants not turning up and dropping out.’ (Research staff, 05).*
    Similarly, research participants stated that understanding what they agreed to was a fundamental responsibility for research participants, as the quote below illustrates:
    *The first role of participants is to know what they are signing up for, what the study is about. That’s very important, so you should know whether it’s risky or not. They must understand what they are signing for and that it is their responsibility to do so. (Study Participant NCT, FGD)*
    Research staff mentioned that they should emphasise more often that their participation to the end of the research is a significant responsibility, as dropping out early by participants can affect the study’s findings. They believe that, in conversations with participants, it’s also important to discuss aspects of their autonomy, such as personal responsibility, knowledge, and information, which will encourage them to stay in the study for as long as possible, if that’s their preference.
  ii. Responsibilities are dependent on the research participant.
    Research staff stated that research participants must take responsibility because they have chosen to participate in the research. They felt it was the responsibility of study participants to ensure they understood what they agreed to and to meet their end of the bargain once they accepted and consented to participate in the research. They said it relies on the study participants to ensure they know what they agree to when they decide to volunteer in research; they should know and understand the type of study they are joining, as well as the risks and benefits associated with it.
    *‘Research participants have a huge role in research studies because the participants, their kids, mothers, or relatives go into the study. So, their role is to ensure they know what will happen to their lives because it is their lives. They need to know what will happen to them after the research study. If the study concerns drugs, they need to have drug compliance; it is their responsibility to have it because that will help them, not the researcher, but themselves; it is their health.’ (Research staff, 02).*
    *‘They need to know that the environment where the research study is taking place is safe, they need to be handled in a safe environment, and it is their responsibility to know that whatever is happening there will be kept confidential because they are people, they have got their rights, they have got their own private life. So, they must know what will happen and everything in that study. And it is their responsibility to know that they have completed the study and need to complete it because if they don’t, the results will be of low quality.’ (Research staff, 01).*
    Research staff believed that the primary responsibility of the participant was to understand the research study and its safety measures, which the research staff could explain. They believe participants understand their commitment only when they comprehend the study. Researchers believed that meeting study visits, even if participants had signed the consent form, also played an essential role once they were enrolled in the study. They think that by consenting to participate in the study, they must also agree to specific study procedures.
    *‘Once they are enrolled in the study, let’s say if it’s a study where it has follow-up visits, either actively where we go to them or passively where they come to us, I think meeting those visits if they have signed the consent form is also important on their end. I think there are study procedures that, if they agree to, they must be part of those procedures.’ (Research staff, 06).*

## Discussion

This is the first study in Malawi to investigate participants’ understanding of their responsibilities in research within Blantyre and Chikwawa districts. We explored research participants’ understanding of their responsibility in clinical research. Our discussion will interrogate this by focusing on the relational responsibilities, expectations, and realities.

### Relational responsibilities from the perspectives of participants

An interesting finding in this study is the variation in understanding of responsibilities by research staff and participants. It is noted that the views of research professionals are likely to differ from those of the public regarding research participation [12]. While research staff are inclined to limit participants’ responsibilities regarding study functionality within the confines of the study design and protocol, participants, on the other hand, take their role in research seriously, as they perceive their involvement as extending beyond the study requirements. They act as advocates of researchers when opportunities arise, dispelling rumours, and encouraging others to volunteer in research after having gone through it themselves.

The motivation for advocacy is mainly based on their experience, perception of the benefits, and immediate individual benefits. This finding aligns with a study where peer experience played a role in encouraging others to participate in resistance training [13]. Findings illustrated that occasionally, researchers and participants shared the same perspective on participants’ responsibilities, for example, comprehending research. However, research participants placed remarkable emphasis on relational responsibilities.

It is worth noting that the literature search did not produce any research publications on participants’ views about their responsibility in research. However, the review managed to yield three articles that listed the responsibilities of research participants [12, 14, 15]. Compared to our findings, the responsibilities mentioned in these articles differ because our research indicates that participants perceive their responsibility in research as more relational. Participants placed great emphasis on relational responsibilities, which they believe are crucial in maintaining working relationships with research staff and the resultant sense of worth that this confers on them.

Participants regard themselves as representatives of researchers in the community by assuming a responsibility to motivate others to join research and supporting researchers in dispelling rumours surrounding research. They perceive themselves as representatives and thus dispel rumours to clear the way for the research they are participating in and for researchers in general. They consider it a contribution to research and a motivator for others to participate in research.

This study discusses responsibilities, and as the findings show, the responsibilities of research participants do not end with the research contract from which these functional responsibilities are derived; rather, they extend beyond this contract. By dispelling rumours, participants cultivate a positive research atmosphere and perception of research within their circles. Nevertheless, the ethical implications of this conduct may warrant further examination. It is essential to note that participants may not always be able to influence others in the community to volunteer in research, especially in urban settings where factors such as societal context and the level of engagement among community members may impact the possibility of encouraging others to participate.

### Functional responsibilities from the perspective of research staff

We define functional responsibilities as responsibilities that directly contribute to study operations and are fundamental for study functionality. The findings of this study demonstrate that the responsibility of participants, as perceived by researchers, is largely based on their understanding of responsibility (17). There are various responsibilities associated with being a research participant. In our findings, some of the responsibilities mentioned included fulfilling study visits, providing biological samples, adhering to medication, being committed to the study requirements, and following other instructions provided by the study staff. These findings align with the research participants’ responsibility as mentioned in [12]. Participants who do not adhere to medication, for example, may put their lives at risk if participating in clinical trials [12, 16]. Furthermore, participants who fail to attend study visits may invalidate the results [16].In addition, the participants’ responsibilities mentioned above align with those outlined in three publications [12, 14, 15].

It is worth noting that during the literature review, we did not come across any research publications that interviewed researchers for their perspectives on the responsibilities of research participants. Another responsibility that both research participants and research staff shared was understanding the research, including its risk factors, procedures, and potential benefits. This is a very essential responsibility, as participants who do not understand the study they are taking part in and all accompanying processes invalidate the informed consent (18). Our findings demonstrate that researchers emphasise the one-time contact when they engage with the participant in a conversation, sharing information during the consenting process and asking participants to ask questions [12]. If the participant comes to ask questions later, the information may not be provided to them [2]. The participant–researcher relationship is fundamentally contractual [17]. For the researcher, it is a functional contract; however, the elements conclude with the informed consent process. Yet, to the participant, it is a relational contract that stretches beyond informed consent.

While researchers acknowledged the importance of participants fulfilling functional responsibilities such as remaining in the study, less attention was given to the relational aspects of engagement that participants emphasised. This suggests a potential gap between how staff and participants understand responsibilities, with implications for how participant–researcher relationships are ethically framed. It is, hence, imperative for researchers to place greater value on and recognise this because it may inform ethical considerations relating to participant-researcher terms of engagement.

### Expectation versus reality in practice

i. Reciprocity of the participant-investigator relationship
  The relationship between research staff and the participants is interdependent. While participants may feel they are helping research staff, researchers rely on participants to collect data to achieve the study’s objectives. This interdependence is evident in the study participants, as most respondents indicated that their role in the research is to help researchers achieve their objectives. They assist researchers in exchange for direct and indirect benefits. For participants in medical care, learning about their health through quick diagnosis and thorough explanations from researchers is not considered a researcher’s obligation. However, receiving this help makes them feel valued [2]and hence lean towards building and maintaining relationships with researchers. Developing close relationships with researchers is considered a positive aspect of the research experience [2] Researchers, however, must clarify that the relationship with participants is reciprocal because researchers draw on the relationships they establish with research participants to promote the functional responsibilities, recruitment, and retention of participants in the study.
ii. There is a disconnect in perceptions.
  There is a distinct variation in perceptions between researchers and participants on what is regarded as the responsibilities of participants. From the researcher's point of view, participants' duties depend on the information they are given when they join the research. This means that there are no standard responsibilities for participants. They vary according to the study design, protocol, and study requirements. On the other hand, participants believe their roles are to encourage others to participate in research, dispel myths surrounding research and help researchers.
  These are self-assigned or assumed roles based on the research participants acknowledging that they were not informed of their roles during the interface with research staff at enrollment. This also means that the responsibilities are not stipulated in the participant information leaflet and thus are not included in the informed consent process, as this is the time when research staff provide information to participants. Participants feel indebted to researchers for the perceived help they received during participation and trust that encouraging others to participate in research is a way of paying back, in an attempt not to disappoint the researchers. This perception inconsistency needs to be addressed by engaging research staff and participants to foster a mutual understanding of participant responsibilities.
iii. Limitations of the Informed Consent Process
  CIOMS, the Declaration of Helsinki and other standard guidelines stipulate the critical elements of informed consent, which are typical for every type of research design. However, these key elements need to include the general responsibilities of participants. As part of the informed consent process, the participant's information leaflet outlines what the protocol expects from participants, including the number of visits, samples, and volumes to be collected during the study. Among the key elements is the right to withdraw; no other right is mentioned.
  We must discuss responsibilities and vice versa to talk and emphasise rights. It is only when participants understand their rights that they can comprehend their responsibilities. While informing participants of their rights, researchers also need to educate participants on the researchers' obligations; this may prevent research participants from having unrealistic expectations about the research process. This way, participants can understand their responsibilities, rights, and duties. This, in turn, will help participants protect themselves as they choose to participate in research.

### Recommendations

#### Rethinking community/participant engagement

Research should be considered as a partnership between researchers and participants. Participants need to be informed of their contribution to research, considering their perceptions of responsibilities in research. They enter this partnership unaware of their duties. Rather than holding participants accountable for non-adherence to study appointments and loss to follow-up, the aim should be to empower participants and inform them of their role and contribution to the research, as well as their rights and responsibilities. This information should be communicated to them before they interact with researchers. This will ensure that participants are empowered and aware of their obligations, ultimately leading to better adherence to study appointments and improved retention. An informed participant will be able to understand their role and responsibility when they decide to participate in research.

When participants are invited to participate in research, they should already be aware of their rights, roles, and responsibilities, including the researcher’s duties and obligations. There is a need to develop general responsibilities of research participants that are standard and applicable to all research designs. Rather than relying solely on the informed consent process, we need to ensure that participants are aware of these details before meeting with research staff. Community engagement should, therefore, include sensitising communities on their rights and responsibilities in research. As noted earlier, during literature review, we did not come across research publications which interviewed researchers for their perspectives on the responsibilities of research participants, therefore more research should be done on responsibilities of research participants in research.

### Study limitations

This study was conducted in the context of the emergence of COVID-19, which prompted the local ethics committee and the Ministry of Health to issue guidelines, restrictions, and recommendations for conducting research studies and for interactions between healthcare workers and the community. The prevalence of COVID-19 also meant a reduction in the number of studies running at that time, thus restricting the number of participants and studies from which to recruit. As a result, the findings from this study may need to be more transferable in specific contexts.

## Conclusions

This study focused on exploring what participants understood as their responsibilities in research and whether their understanding differed from the researchers’ perceptions. The difference in perceptions concerning participant responsibilities needs to be aligned by engaging research staff to develop transparent relationships. Research participants should be aware of their rights and duties before interacting with researchers. Researchers should strive to find effective ways to communicate responsibilities to participants, thereby achieving a mutual understanding.

### Implications for future research

This study provided insight into the roles and responsibilities of research participants from their perspectives. It brought out the discrepancies between the views of research staff and research participants. Future research needs to explore how best to communicate research participants’ responsibilities and delve further into the complexity of the participant–investigator relationship, including when or how the research contract should be terminated. Furthermore, future research should investigate which specific rights and responsibilities should be incorporated into the informed consent process.

## Supplementary Information

Supplementary Material 1.

Supplementary Material 2.

Supplementary Material 3.

Supplementary Material 4.

## Data Availability

Data is available from the corresponding author upon reasonable request.

## Abbreviations

CIOMS: Council for international organisation of medical sciences
APA: American psychological association
QECH: Queen elizabeth central hospital
CK DHO: Chikwawa district hospital
IDIs: In-depth interviews
FGDs: Focus group discussions
CT: Clinical trial
Non-CT or NCT: Non-clinical trial
PI: Principal investigator
COMREC: College of medicine research ethics committee
LSTM: Liverpool school of tropical medicine
DHO: District health office
