# Supplementary material for: Participant and researcher understandings of research responsibilities in malawi: a comparative analysis
Source: BMC Med Ethics. 2025 Oct 24;26:147. doi: 10.1186/s12910-025-01306-1 (PMC12551194; doi:10.1186/s12910-025-01306-1)
Supplement: Supplementary file 1 — Supplementary Material 1. [file 12910_2025_1306_MOESM1_ESM.docx]

**
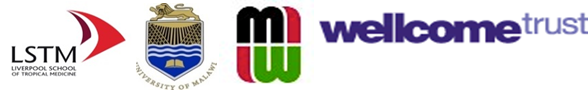
**

**Appendix 1**

**Individual participants topic guide**

**Exploring understanding of research participants on their roles and responsibility in clinical research in Southern Malawi: Expectation vs reality in practice**

**Personal details**

Please tell me where you stay and How old are you?

How far did you go with education? What do you do for a living?

**Research experience and views on participants’ responsibilities**

Please tell me the details of the research study (ies) you are involved as a research participant?

Why did you decide to become involved in these research studies? Some people say that people join studies to be altruistic and to help your community. What do you think?

Please tell me what you think your roles and responsibilities are in research? How has that impacted (or not) the decision to join research?

How and at what stage were you informed of your roles and responsibilities in research? Were you satisfied/ not satisfied with the process and the information you received? What could be improved?

Tell me what actually happened; what is your overall experience of being involved in these studies and how has it played out?

What does it mean to be a research participant to you?

***If you were not informed of your roles and responsibilities;***

What other things do you understand on research participation? From where did you get this information?

Were there any disadvantages of not being informed of your responsibilities? Please explain

What would necessitate your understanding of the roles and responsibilities in research?

**Views research practice**

Do you feel protected as a research participant while you are participating in the study? How protected do you feel? What should be done to protect you better?

Do you think there should be principles to guide research participants conduct while they are participating in the study?

**THANK YOU**
