# Supplementary material for: Participant and researcher understandings of research responsibilities in malawi: a comparative analysis
Source: BMC Med Ethics. 2025 Oct 24;26:147. doi: 10.1186/s12910-025-01306-1 (PMC12551194; doi:10.1186/s12910-025-01306-1)
Supplement: Supplementary file 2 — Supplementary Material 2. [file 12910_2025_1306_MOESM2_ESM.docx]

**
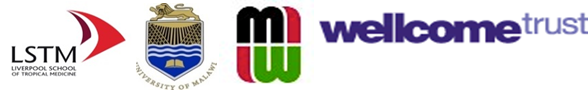
**

**Appendix 2**

**Researcher topic guide**

**Exploring understanding of research participants on their roles and responsibility in clinical research in Southern Malawi: Expectation vs reality in practice**

**Research Experience and views on participants’ roles and responsibilities**

Tell me about your research experience and the kinds of studies you are involved in.

What do you feel drives research participants into participating in research?

Generally, what is the overall experience of being involved in these studies?

Please tell me what you think are the roles and responsibilities of participants in research?

How and at what stage do you inform your participants of their roles and responsibilities when they decide to participate in research?

How do you handle the situation when the participant doesn’t understand their roles or has different expectations?

***If you do not inform your participants of their roles and responsibilities.***

Were there any disadvantages or advantages of not informing them?

Is there anything you would change now or do differently?

How best can you inform participants of their roles and responsibilities?

**Views on research Practice**

Do you think there should be principles to guide research participants conduct while they are participating in research?

**THANK YOU**
