# Supplementary material for: Participant and researcher understandings of research responsibilities in malawi: a comparative analysis
Source: BMC Med Ethics. 2025 Oct 24;26:147. doi: 10.1186/s12910-025-01306-1 (PMC12551194; doi:10.1186/s12910-025-01306-1)
Supplement: Supplementary file 3 — Supplementary Material 3. [file 12910_2025_1306_MOESM3_ESM.docx]

**
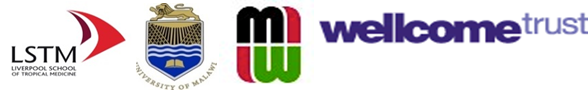
**

**Appendix 3**

**Focus Group Discussions topic guide**

**Exploring understanding of research participants on their roles and responsibility in clinical research in Southern Malawi: Expectation vs reality in practice**

**Views on participant’s roles and responsibilities**

1. Why do people decide to become involved in these research studies?
2. What do you think are the roles and responsibilities of participants in research?

**Probe**: How do the roles impact (or not) the decision to join research?

1. Is it important for research participants to know and understand their roles and responsibilities in research?

**Probe**: why do you think or not think so? Explain

1. How can knowledge and understanding their roles and responsibilities impact research practice?
2. At what stage of the research should participants be informed of their roles and responsibilities?
3. What do you think can necessitate participants understanding their role and responsibilities?

**Views research practice**

1. Do you think there should be principles to guide research participants conduct while they are participating in the study? If so, Why? or Why not?

**THANK YOU**
